# Pregnant Women’s Experiences of Stress During the COVID-19 Pandemic: A Qualitative Study

**DOI:** 10.3390/healthcare14010014

**Published:** 2025-12-20

**Authors:** Chinyere N. Reid, Abraham Salinas-Miranda, Cheryl Vamos, Kimberly Fryer Segro, Jason Beckstead, William M. Sappenfield

**Affiliations:** 1 College of Medicine, University of Florida, Jacksonville, FL 32209, USA; 2 College of Public Health, University of South Florida, Tampa, FL 33612, USA; asalinas@usf.edu (A.S.-M.); cvamos@usf.edu (C.V.); kef4x@virginia.edu (K.F.S.); jbeckste@usf.edu (J.B.); wsappenf@usf.edu (W.M.S.)

**Keywords:** pregnant women, COVID-19, mental health, stress, psychological, anxiety, depression, qualitative research, pandemics, social support

## Abstract

**Background/Objective:** The COVID-19 pandemic resulted in unprecedented societal changes globally and negatively impacted the psychosocial health of pregnant women. This study aimed to explore how direct, indirect, and unrelated factors associated with the COVID-19 pandemic influenced stress levels among pregnant women. **Methods:** This interpretivist qualitative study employed open-ended survey questions to capture the lived experiences of 313 pregnant women in the third trimester residing in Florida between January and March 2022, during the Omicron surge. Thematic analysis was conducted, guided by the Stress and Coping Theory and the Stress Buffering Theory. **Results:** Participants described a range of stressors directly and indirectly related to the COVID-19 pandemic that affected pregnant women. Themes related to increased stress were (1) fear, worry, and anxiety related to COVID-19 infection, (2) fear, worry, and anxiety related to preparedness for birth/baby due to pandemic restrictions, (3) prevention concerns associated with COVID-19, (4) lack of social support, (5) return to normalcy, (6) health-related social needs, (7) physical health issues, and (8) navigating conflict and grief. Conversely, stress-reducing factors were (1) preventive measures during the pandemic, (2) coping strategies, (3) not having to work, and (4) social support. **Conclusions:** Pregnant women experienced heightened stress due to a complex interplay of factors related directly or indirectly to the COVID-19 pandemic. It is important that maternity care, mental health and health-related social needs screenings and referrals, and tailored interventions are integrated into public health crises preparedness plans to limit the stress that pregnant women experience and support their well-being.

## 1. Introduction

In early 2020, COVID-19 was declared a global pandemic by the World Health Organization [1] and led to widespread lockdowns, strain on the healthcare system, and economic instability [2]. As a result, many in the general public experienced unprecedented uncertainties and mental health challenges [3]. This was especially true for pregnant women who faced the fear of infection and the added impact on their pregnancy, issues with prenatal care access, and limited social support [4,5].

Since the COVID-19 pandemic, there has been a significant increase in the prevalence and severity of stressors experienced by pregnant women affecting their mental health [6,7,8]. Several studies have been conducted on the stress experienced by pregnant women during the pandemic. Zilver et al. [9] reported that pregnant women from the Netherlands scored significantly higher on the Perceived Stress Scale (PSS-10) during the pandemic compared to those before the pandemic. In a national survey of pregnant women across all 50 U.S. states, Preis et al. [10] found that almost a third of their sample of 4451 pregnant women reported higher stress due to the pandemic. This increased stress during the pandemic has been linked to an increase in mental health conditions in pregnant women. In another U.S.-based study of 725 pregnant women living in San Francisco, California, subjects reported higher COVID-19-related stress, and this was significantly associated with higher depressive symptoms [11]. Thayer et al. [12] found that COVID-19-associated financial stress was associated with more than twice the risk of depression during pregnancy.

Pregnancy is considered a vulnerable period during which stress is increased and external stressors can increase psychological distress and the risk of development or worsening of preexisting mental health issues [13,14]. Prior to the pandemic, perinatal mental health was considered a public health concern, with a global prevalence among pregnant women of 20.7% for prenatal anxiety and 22.9% for prenatal depression [15,16]. However, since the pandemic, several systematic reviews have reported increased prevalences of anxiety between 30.5% to 42% and 25.6% to 30.0% for depression during pregnancy [17,18,19]. Anxiety and depression have been linked to adverse maternal and fetal outcomes, including preterm birth, low birth weight, attachment difficulties, developmental delays, postpartum depression, and suicidal ideation [20,21,22].

Globally, qualitative and mixed-methods studies have reported increased stress among pregnant women during the pandemic. For instance, one scoping review and several international qualitative studies from Canada, Ireland, Iran, Turkey, and the UK found key themes of pregnant women experiencing increased psychological distress, isolation, limited social support, and disruptions in prenatal care [23,24,25,26,27,28]. However, these studies captured pregnant women’s experiences earlier in the pandemic, before the implementation of COVID-19 vaccines and other variant surges. Therefore, exploration of pregnant women’s experiences of stress during the later phases of the pandemic remains limited globally. In addition, the increases in mental health issues are unlikely due to the result of a singular event but rather driven by a combination of stressors directly or indirectly related to the COVID-19 pandemic. Despite a growing body of literature to date on the mental health impacts of the pandemic on pregnant women, no known studies have utilized a theory-based approach to qualitatively explore direct and indirect COVID-19 factors that affected stress among pregnant women living in Florida, U.S. Furthermore, most of the qualitative literature in the U.S. has focused on earlier periods of the pandemic [25,29,30,31,32], often missing the ever-changing context of later COVID-19 waves. This study addresses these gaps by exploring the lived experience of pregnant women during a unique period and time of uncertainty—the Omicron-dominant period (beginning December 2021) [33]—to inform future preparedness for public health emergencies.

Using a theory-guided approach, this study was informed by the Stress and Coping Theory and the Stress Buffering Theory. The Stress and Coping Theory postulates that individuals’ response to stress is dependent on how they appraise stressful events, and this in turn evokes coping strategies in an attempt to manage these stressors [34]. The Stress Buffering Theory suggests that social support mitigates or “buffers” the harmful effects of stress on mental well-being [35]. Both theories were selected because they helped inform this study’s exploration of how pregnant women processed and responded to direct and indirect stressors and the role of factors such as coping strategies and social support that may have increased and decreased their stress experienced during the pandemic.

Therefore, the aim of this qualitative study was to explore how pregnant women experienced direct and indirect COVID-19 factors that increased or decreased their stress during the pandemic. The research questions were (1) What factors directly related, indirectly related, or unrelated to the COVID-19 pandemic increased stress among pregnant women? and (2) What factors directly related, indirectly related, or unrelated to the COVID-19 pandemic decreased stress among pregnant women?

## 2. Materials and Methods

### 2.1. Study Design

This primary qualitative study assessed direct and indirect COVID-19 factors impacting stress among pregnant individuals during the pandemic. Data collection and primary qualitative analysis were conducted on two open-ended questions that were embedded in a larger cross-sectional survey. The parent survey examined the influence of stress, social support, and coping on the anxiety, depression, and maternal-fetal attachment of pregnant women during the COVID-19 pandemic and consisted of validated psychometric scales measuring each domain [36]. Although the qualitative component of the study was embedded within a larger quantitative survey, an interpretivist qualitative approach was used to analyze narrative responses to understand pregnant women’s interpretations of their experiences and factors that impacted their stress during the pandemic. Data collection occurred from January to March 2022, during the COVID-19 Omicron variant wave in Florida. The study was approved by the University of South Florida (USF) Institutional Review Board.

### 2.2. Eligibility Criteria

Eligible participants were at least 18 years old, in their third trimester of pregnancy (28–42 weeks gestation), residing in Florida at the time of the study, and able to read and speak English. Participants were excluded if they were surrogates or intended to give up their baby for adoption at birth.

### 2.3. Participant Recruitment and Enrollment

Participants were recruited by the primary researcher (CR) at two prenatal clinic sites in Florida, the USF Health Obstetrics Clinic, and the TGH Genesis Women’s Center at Healthpark. Potentially eligible participants were identified through a review of electronic medical records, considering maternal age, gestational age, and appointment time. Identified participants were approached by a certified medical assistant (CMA) or their healthcare provider and introduced to the study. The primary researcher introduced themself and provided interested patients with detailed information about the study purpose, survey duration, and participation requirements.

Upon verbal agreement to participate, patients gave informed electronic consent at the beginning of the questionnaire, then completed the anonymous online survey questionnaire on a provided tablet. Participants received a $10 Amazon gift card via email for their time. Of the 408 participants who initiated the questionnaire, five respondents were excluded because of unrealistic short completion times or implausible response patterns, resulting in a total of 403 participants that were included in the final overall survey analysis.

### 2.4. Survey

The qualitative portion of the anonymous survey questionnaire included two optional open-ended questions: (1) “Please tell us about any factors in your life that you believe increased your stress while pregnant during the COVID-19 pandemic. These factors could include certain people, circumstances, or other experiences,” and (2) “Please tell us about any factors in your life that you believe decreased your stress while pregnant during the COVID-19 pandemic. These factors could include certain people, circumstances, or other experiences.” Additionally, the survey included demographic questions on maternal age, race, ethnicity, education level, marital status, gravida, pregnancy wantedness, COVID-19 vaccination status, and vaccination intention for those currently not vaccinated. The full survey instrument was initially pilot tested on six people prior to the start of data collection.

### 2.5. Analysis

The research team consisted of five doctoral-level public health researchers and one clinician specialized in obstetrics and gynecology. The researchers were trained in both qualitative and quantitative research methodologies with experience in maternal and child health topics. A thematic analysis was conducted on the open-ended survey responses, following Braun and Clarke’s six-phase approach [37], and multiple strategies were used to enhance trustworthiness. The primary researcher (CR) reviewed all responses and developed an initial codebook, incorporating a priori structural codes based on the Stress Buffering Theory and Stress and Coping Theory, as well as emergent codes. After a second review, additional emergent codes were incorporated into the codebook. A doctoral research assistant with expertise in thematic analysis served as a second coder. Both the primary researcher and doctoral research assistant discussed the coding process and coded approximately 10% of the qualitative responses [38]. Two coding cycles were conducted, during which both researchers resolved coding discrepancies and iteratively refined the codebook to achieve consensus, reaching an inter-coder reliability of at least 80% (k = 0.83) [39]. The primary researcher coded the remaining participant responses. Coding was performed using MAXQDA 2020 (VERBI Software, Berlin, Germany), and the coded data were then analyzed using a thematic approach [40]. An audit trail was kept of all coding decisions and development of themes. CR remained mindful of the power dynamics inherent in research and sought to honor the voices of participants by allowing their words to guide the emergence of themes. CR also engaged in peer debriefing to review interpretations of the data. Data saturation was achieved when no new themes emerged from the data [41] and occurred before all coding was complete. However, coding continued until all responses were analyzed to ensure that all participant experiences were captured and confirmed that no new themes were identified. Demographic questions were analyzed with descriptive statistics in SAS software, version 9.4 [42].

## 3. Results

Of the 403 pregnant women included in the survey, 313 completed the qualitative portion. Participant characteristics are described in Table 1. Participants were on average aged 30.4 years (SD 5.5). The majority self-identified as White (66.8%), non-Hispanic (69.3%), completed high school as their highest level of education (36.1%), and were married or in a committed relationship (86.9%). Most were multigravida (62.3%), reported wanting their pregnancy now (78.0%), and were fully vaccinated for COVID-19 (53.4%). Of those who were unvaccinated, most had no intention to be vaccinated (78.8%).

Several major themes directly and indirectly related to COVID-19 were identified that were reported to have contributed to the increase and decrease in maternal stress in pregnancy during the pandemic. Direct COVID-19-related factors were specifically linked to the pandemic itself. Indirect or unrelated COVID-19 factors were not directly caused by the pandemic but may have influenced stress levels during that period.

### 3.1. Direct COVID-19-Related Factors Increasing Maternal Stress

Several identified key themes of direct COVID-19-related factors that increased stress among pregnant participants were (1) fear, worry, and anxiety related to COVID-19 infection, (2) fear, worry, and anxiety related to preparedness for birth/baby due to pandemic restrictions, (3) prevention concerns associated with COVID-19, (4) lack of social support due to the pandemic, and (5) return to normalcy. Participant quotes related to each theme are shown in Table 2.

#### 3.1.1. Fear, Worry, and Anxiety Related to COVID-19 Infection

Participants frequently described their fears of contracting COVID-19 infection and its health impact on them, their unborn baby, or other people around them, and this increased their stress during the pandemic. Participants shared that these fears limited their involvement in daily activities, social interactions, and in some cases made them hesitate to seek necessary care when needed. Many participants who identified as healthcare workers expressed particularly high levels of concern about workplace exposure and potential transmission of COVID-19 infection to their families. Although some participants previously recovered from COVID-19 infection, they described how “the thought of getting COVID-19” reinfection and its effects on their current pregnancy was stressful.

*I’m a nurse. The pandemic has added so much stress…[the thought of] getting COVID-19 and losing the baby has been stressful…we have had so many patients turn up with positive results and exposing us. I have a child at home.*

[391]

#### 3.1.2. Fear, Worry, and Anxiety Related to Preparedness for Birth/Baby Due to Pandemic Restrictions

The pandemic-related restrictions caused significant anxiety regarding birth preparedness. Many participants shared that they were worried about changes to their birth plans, hospital capacities, and the potential lack of a support person being present during childbirth. Some described how experiences and stories of traumatic births early in the pandemic further increased these concerns. Additionally, some participants felt anxious about post-delivery separations from their newborns. Although concerns about not receiving prenatal care were rarely mentioned, several participants expressed feeling “worried” about the quality of postpartum care they would receive during the pandemic.

*…According to the new TGH policy, there will only be one support person allowed in the room. I was hoping to hire a doula for additional support to achieve an unmediated birth, but now that doesn’t seem possible and has slightly increased worry surrounding how the birth will go.*

[115]

#### 3.1.3. Prevention Concerns Associated with COVID-19

Prevention concerns emerged as a major stress factor and comprised two subthemes: (1) concerns related to COVID-19 mandates and (2) concerns related to COVID-19 vaccination.

Concerns Related to COVID-19 Mandates: This subtheme overlapped with the major themes fear, worry, and anxiety related to COVID-19 infection and fear, worry, and anxiety related to preparedness for birth/baby due to pandemic restrictions. Participants additionally expressed feelings of distress over inconsistent adherence to safety measures by the public and restrictive hospital policies that might affect their labor and delivery experiences. Some participants described feeling “uncomfortable” and “frustrated” about safety mandates such as wearing face masks, hospital policy allowance of a support person in the delivery room, and the potential separation from their baby at birth, which they explained further increased their stress. Some participants shared their belief that wearing a mask during childbirth was unnecessary because it hindered communication, and some described taking a COVID-19 test as “extremely uncomfortable”.

*I feel mandates are unnecessary and create stress. I hate being forced to wear a mask when it is not actually effective. It ruins communication. I’m worried I will be forced to take an extremely uncomfortable COVID-19 test in order to not wear a mask during labor.*

[393]

Concerns Related to COVID-19 Vaccination: Many participants described feeling stressed about COVID-19 vaccination. They reported that they were concerned about the safety of COVID-19 vaccines and conflicting opinions about it, which they said influenced their vaccination decision during pregnancy. A few participants described feeling pressured by healthcare providers and their employers to get vaccinated. Some vaccinated participants emphasized the worry and stress they experienced when they were surrounded by individuals who were not vaccinated and described how this was sometimes a source of family conflict.

*Getting out of the military at the same time of my due date is stressful and being almost kicked out or reprimanded for refusing the COVID-19 vaccine while pregnant has been very stressful, as well. My workplace is a really stress induced environment.*

[40]

#### 3.1.4. Lack of Social Support Due to the Pandemic

Many participants expressed that they lacked the social support they needed during pandemic. The main sources of support they described were from a significant other, family, and friends. Participants shared that especially when they were sick, sources of support were at times unavailable to provide mostly instrumental support (e.g., childcare, household chores, getting to appointments) and emotional support (e.g., feeling loved, someone to listen). Most participants expressed that the need to self-isolate, restrictions on social interactions, and travel constraints impeded the support that they desired during pregnancy, childbirth, postpartum, and recovery. They reported that this led them to feel increasingly stressed during the pandemic.

*Because of COVID-19 a lot less family and friends come around in general or even to help with my toddler when I am sick or in pain and it has been hard since many people work from home and do not like going out unless it’s necessary. This also makes it difficult to make it to certain appointments when I’m not able to drive on days I am really nauseous or in pain.*

[397]

#### 3.1.5. Return to Normalcy

A few participants expressed that their uncertainty about the future and a strong desire to return to pre-pandemic normalcy were significant stressors for them.

*Something that has increased my stress while pregnant during the COVID-19 pandemic is not knowing when everything will be back to normal and/or if this is something we will live with the rest of our lives.*

[3]

### 3.2. Direct COVID-19-Related Factors Decreasing Maternal Stress

Major themes of direct COVID-19-related factors that decreased stress among pregnant participants during the pandemic included (1) preventive measures during the COVID-19 pandemic, and (2) pandemic coping strategies. Participant quotes related to each theme are shown in Table 2.

#### 3.2.1. Preventive Measures During the COVID-19 Pandemic

Many participants stated that they experienced stress relief because of COVID-19 precautionary behaviors and people being vaccinated which made it safer and “easier to socialize and have a better support system”. They described behaviors such as others adhering to wearing a mask, sanitizing hands, social distancing, COVID-19 testing, as examples. Many participants shared that they welcomed the chance to shelter-in-place and work from home to reduce their risk of contracting COVID. Surprisingly, although many participants said they considered the restriction on the allowance of a support person in the delivery room as stressful, one participant acknowledged that “I don’t want them there. Having less visitors allowed in the delivery room is helpful for me”.

*Not having to go into work/an office has been really great, especially since I was so sick the first trimester. Being able to work from home has definitely decreased stress, also in knowing I’m much less exposed [to the COVID-19 virus]*.

[206]

#### 3.2.2. Pandemic Coping Strategies

Participants described various coping strategies that they used to manage and decrease their stress. Participants primarily described using avoidance coping strategies such as “staying away from news” focused on the pandemic and “distracting” themselves with activities. Additionally, many participants shared that they avoided interactions with people whose COVID-19 beliefs and behaviors negatively affected their emotions.

*Spending more time indoor with family because I get more help or spend time doing things to get distracted.*

[169]

### 3.3. Indirect or Unrelated COVID-19 Factors Increasing Maternal Stress

Although factors directly related to the pandemic contributed substantially to the stress experienced by pregnant women, participants identified the following themes as indirect or unrelated COVID-19 stressors: (1) lack of social support, (2) health-related social needs, (3) physical health issues, and (4) navigating conflict and grief. Participant quotes related to each theme are shown in Table 3.

#### 3.3.1. Lack of Social Support

Similarly to direct COVID-19-related stressors, participants identified a “lack of” social support as a stressor unrelated or indirectly related to COVID-19. Some participants expressed that this was especially true when a support person(s) was unavailable to assist either because they resided in a different state or they were deployed in the military at the time, and not necessarily because of the pandemic. In some cases, participants described instances where they experienced challenges in a relationship with a support person or a support person was struggling with mental health issues that hindered them from providing the support they needed. While these factors were either indirectly or unrelated to the pandemic, participants felt that they contributed to their heightened stress.

*I’ve had a lack of direct family support due to family being out of state, and the only family present has mental health issues that cause them to be violent. So, this has caused me at times to feel less support…*

[66]

#### 3.3.2. Health-Related Social Needs

Some participants described their experiences of health-related social needs which they felt were a source of increased stress because they believed that it added to the burden of keeping up with the rising cost of living during the pandemic. These participants said that difficulty with finances from lost income, reduced work hours, unemployment, and a lack of paid maternity leave were stressful for them. One participant shared that she felt stressed because “my hours at work have been dropping, not having money to take care of the bills that are coming up, and no resources to really help me. No babysitter, nothing!”, while another felt that it was “harder to find work during the pandemic, especially while pregnant.” Healthcare workers also reported that they felt stressed because of adverse employment conditions such as “increased workload, staff shortages and increased work demands from an influx of sick patients needing care”. Other factors that participants said were extremely stressful during the pandemic included unstable housing situations, difficulty in accessing affordable mental healthcare, and the continuity and quality of maternity care, especially after relocating to a new state.

*…access to mental healthcare that is affordable has become more difficult and scarcer…Utilizing health insurance and benefits has become more difficult as the system seems to be overloaded…*

[372]

#### 3.3.3. Physical Health Issues

A few participants highlighted that their physical health issues related to pregnancy were a stressor because it affected their ability to perform daily life activities. A common example given was dealing with pelvic pressure and pain. For one participant this made her feel anxious and she shared that “Grocery shopping with my 11-month-old while being nine months pregnant with pelvic pressure can cause me anxiety and my shopping trips stay short”. In addition to dealing with personal health issues, participants expressed that they were highly stressed if their unborn baby was diagnosed with a health ailment.

*This is my sixth child, so my muscles are not as strong, and it hurts to walk. This is stressful when trying to work and complete tasks.*

[33]

#### 3.3.4. Navigating Conflict and Grief

Navigating conflict and grief emerged as a stressor. Some participants talked about being involved in disputes with family members or a significant other. A few others mentioned that their experience of grappling with the loss and grief of a loved one further worsened their stress.

*Dad passed away. First grandkid and a boy which is what he always dreamed of. Bittersweet feelings. Sometimes husband doesn’t understand the time limitations and does not contribute to preparing for the journey or think things through.*

[367]

### 3.4. Indirect or Unrelated COVID-19 Factors Decreasing Maternal Stress

Themes identified as indirect or unrelated COVID-19 factors decreasing stress included: (1) not having to work, (2) coping strategies, and (3) social support. Participant quotes related to each theme are shown in Table 3.

#### 3.4.1. Not Having to Work

Some participants mentioned that not having to work during pregnancy was a stress-reliever during the pandemic. Participants emphasized that staying at home enabled them to have “more time” with family and “be available to care for my family”. Additionally, they expressed that this newfound time at home gave them the chance “to focus on self-care and health” activities like exercising, enjoying nature, practicing yoga, and engaging in various activities that improved their overall well-being during the pandemic.

*Having more time with members of my household has been helpful to decrease stress. I have taken more time being home and doing projects and home to pour into the house and the family that lives there.*

[372]

#### 3.4.2. Coping Strategies

Participants shared positive coping strategies they frequently used to lessen stress during the pandemic. Respondents, especially those who had prior childbirth experience, described participating in activities in preparation for their newborn’s arrival. They talked about relying on their faith and faith community, praying or seeking prayers, and active involvement in church activities also helped curb stress. One participant said, “Having a relationship with Jesus, spending time with Him in prayer, reading His Word, and knowing that others are praying for me” helped decrease her stress. For some, they shared that maintaining a positive way of thinking such as “The thought of [baby’s name] coming out healthy and me surviving labor”, surrounding themselves with positive influences, and involvement in activities they enjoyed were strategies that helped them manage and decrease their stress.

*Having a relationship with Jesus, spending time with Him in prayer, reading His Word, and knowing that others are praying for me.*

[392]

#### 3.4.3. Social Support

Social support was also an important indirect COVID-19 factor that reduced stress during the pandemic. A majority of participants described mostly experiencing emotional and instrumental support from family, close friends, and significant others. One participant shared that “My family and friends have been there for me through this pregnancy and have helped me when I get overwhelmed.” Some participants also reported that they found comfort and solace from their pets, coworkers, and healthcare providers. Participants described the emotional support they received as feeling “great joy”, “loved”, “happy”, and “listened to” by others. They mostly described the instrumental support they received in the form of assistance with childcare, which they said offered stress relief.

*Knowing that my mom is doing everything she can to keep me happy, and my siblings and boyfriend make sure I have everything I want and need even when I say I don’t need it or want it.*

[272]

## 4. Discussion

This study explored the direct and indirect or unrelated COVID-19 factors that increased and decreased pregnant women’s stress during the pandemic. Overall, study findings revealed that pregnant women experienced a multitude of direct and indirect or unrelated COVID-19 factors that heightened and decreased their stress. The pandemic was found to be a major source of multiple stressors and protective factors impacting the well-being of pregnant women. These experiences occurred during a unique period of the pandemic—the Omicron variant surge—when evolving public health protocols and vaccine hesitancy were prominent. Findings revealed factors relevant to preparedness for future public health emergencies.

Overall, factors stemming directly from the COVID-19 pandemic that were identified as major stressors to participants encompassed fear and anxiety of contracting COVID-19 infection and its effects on their unborn baby, and factors related to pandemic policies restricting birth plans, support persons, and vaccination choices. This study revealed that pregnant women’s fears and anxiety of contracting COVID-19 infection were not just limited to their own health but also to the health of their unborn baby, mirroring prior studies [6,43]. This study also supports Liu et al.’s [44] findings that pregnant healthcare workers experienced greater psychological distress than pregnant non-healthcare workers because of fear of increased exposure to COVID-19 infection. An association between pregnancy and moderate to high levels of fear of COVID-19 infection has been established in the literature and linked to maternal distress, anxiety, and depression in pregnancy [45,46,47]. COVID-19 fears and concerns extended to changes to birth plans and possible separation from their newborn at birth and resulted in heightened stress. Similarly, studies have shown that these uncertainties about birth plans and experiences during labor and delivery led to increased maternal stress and anxiety and lowered their satisfaction with their childbirth experience [43,48,49]. A systemic review by Tan and colleagues [50] found that primigravida women were more likely to experience higher levels of stress because it was their first pregnancy and a time of uncertainty compared to multigravida women, who were more likely to feel prepared and adapt to changes during the pandemic. This, however, did not hold true for all pregnant women during the pandemic.

Studies have shown that COVID-19 prevention practices such as mask mandates, COVID-19 testing, and social distancing were major stressors for pregnant women [47,51]. Nonetheless, many participants also deemed it a stress reliever during the pandemic because these practices protected them and their family from COVID-19 infection [52]. Additionally, safety concerns and COVID-19 vaccine hesitancy among study participants reflect other studies’ findings that COVID-19 vaccine hesitancy and acceptance among pregnant women were driven by their motivation to protect themselves and their unborn baby [53,54,55]. Therefore, to support informed vaccine decisions, it is important that information is communicated clearly and consistently, misinformation and mistrust are addressed, pregnant patients are reassured, and efficient vaccine rollout strategies are utilized [56].

Social support emerged as a theme directly and indirectly related to COVID-19 that affected participants’ stress. Pregnant women lacked emotional and instrumental support, which they primarily sought from a significant other, friends, and family. Restrictions to having a support person present during childbirth or having assistance with childcare heightened stress. COVID-19-related public health restrictions such as social isolation and travel constraints also limited social support and were found to be major drivers for psychological distress in pregnant women during the pandemic [5,57]. These study findings align with a systematic review by Al-Mutawtah et al. [58], who found that a lack of emotional and instrumental support from social networks negatively impacted maternal stress, well-being, and health behaviors during the pandemic. One study also found that women’s partners were stressed about not being able to support them during prenatal appointments and labor and delivery [59]. However, the few pregnant women who perceived satisfactory social support during the pandemic reported feeling less stress and mental anguish, consistent with previous studies [58,60]. Study findings further support the Stress Buffering Theory [35] of how support buffers stress and highlight the importance of adequate social support and strong social networks for pregnant women during the COVID-19 pandemic and other public health crises.

Despite participants experiencing a multitude of stressors, they frequently engaged in adaptive coping strategies (e.g., participating in activities that brought them joy, relying on their faith and faith community) and maladaptive coping strategies (e.g., avoiding news regarding COVID-19 and interactions with people whose COVID-19 beliefs and behaviors induced psychological distress) to mitigate stress. These findings align with the Stress and Coping Theory [34], where participants’ appraisal of stressful events during the pandemic evoked different coping strategies in an effort to manage stress. Participants’ adaptive and maladaptive coping strategies mirror findings from previous literature of strategies such as preparing for birth, seeking emotional support from close relationships, faith-based coping, participating in indoor activities, and avoidance of the stressor, which were used to manage stress during the pandemic [24,61,62]. A literature review by Cigaran et al. [63] examining the strategies pregnant women used to cope with stress during the pandemic found that pregnant women utilized both adaptive and maladaptive coping strategies during the pandemic. Adaptive coping strategies were found to be more effective in reducing negative psychological consequences of the pandemic compared to maladaptive coping strategies such as avoidance coping, which has been linked to depression [63,64].

Health-related social needs were indirect stressors that increased participants’ stress. Study findings are in line with prior research where pregnant women were found to be disproportionately affected by socioeconomic, housing, employment, and healthcare access challenges [12,65,66,67,68]. In addition, participants being stressed about dealing with their own physical health issues supports quantitative findings reporting a significant negative association between pregnant women’s physical health status and their stress levels during the pandemic [69,70]. Navigating relationship conflicts and grief from the loss of loved ones exacerbated participants’ stress. Pregnant women’s stress from conflicts within personal and family relationships tended to stem from pandemic-related lockdown measures [71]. Studies have found that pregnant women who experienced grief during the pandemic were more likely to have greater psychological distress and thoughts of self-harm [72,73]. According to a systematic review, the prevalence of domestic violence among pregnant women, a form of relationship conflict, increased during the pandemic [74]. Given the exacerbation of health-related social needs, maternal mental distress, and relationship conflicts during the COVID-19 pandemic, it is important that all pregnant women receive screenings and appropriate referrals as well as being reassessed throughout pregnancy for mental health and health-related social needs, including domestic violence. The development of community-based partnerships and referral systems can also aid in further improving access to appropriate behavioral interventions and support within communities during future public health crises [75].

The Stress and Coping Theory and the Stress Buffering Theory both help explain how pregnant women appraised stressors during the pandemic and why certain factors increased or decreased their stress [34,35]. Pregnant women who appraised greater threats during the pandemic, such as an increased fear of contracting COVID-19 infection, worries about birth plans, or concerns about COVID-19 mandates, reported greater stress. On the other hand, those who perceived greater social support or stable resources described experiencing less stress. These theory-informed findings highlight policies that could be implemented into routine prenatal care. Providing pregnant women with resources that help them manage stress and strengthen positive coping skills, delivering readily available resources for support, and providing psychosocial support would be beneficial. Ensuring that pregnant women receive clear and consistent communication about their health risks, maintaining continuity of maternity care, and addressing their social needs may further reduce the stress that pregnant women experience during public health crises [63,76,77].

This study has several research, clinical, public health, and policy implications. To better identify intervention targets, future research should focus on both direct and indirect stressors affecting maternal health during major public health crises. Conducting longitudinal studies across pregnancy trimesters and the postpartum period could also reveal how stressors evolve over time. This study emphasizes the need for clear, consistent, and empathetic patient-provider communication and a patient-centered approach, especially during times of public health emergencies. Improving prenatal care and social support on virtual platforms and integrating routine mental health and health-related social needs screenings and referrals as standard care may help identify and address direct and indirect stressors as a result of public health crises in a timely manner. This study also highlights the importance of creating policies that safeguard the continuity of maternity services, allowing the presence of support persons during childbirth, and providing a clear and streamlined rollout of vaccine communication for pregnant women to lessen avoidable stress. The implementation of policies to protect pregnant workers and allow for flexible leave during infectious disease pandemics may also help alleviate unnecessary stress. Overall, study findings emphasize the importance of integrating comprehensive healthcare for pregnant women into public health emergency preparedness plans to support their well-being.

Trustworthiness of the study was enhanced through multiple strategies: the use of double-coding, codebook refinement through an iterative process, and theory-guided interpretations (confirmability and reliability), thick descriptions (transferability), methodological documentation and audit trails (dependability), and peer debriefing (confirmability) [78].

This study had several strengths. To our knowledge, this is the first study to explore how both direct and indirect COVID-19 factors affected stress among pregnant women in Florida during the pandemic. Data was collected during the Omicron variant wave, a critical period when there were heightened vaccine safety concerns, uncertainty about the future, and public health recommendations were evolving. Data analysis and interpretation of findings were guided and strengthened by incorporating two complementary theories. Additionally, this study had a large and sociodemographically diverse population, which allowed a myriad of lived experiences to be captured.

This study had some limitations. Recruitment was performed at two clinic sites within a single state—Florida—potentially limiting generalizability of findings to other clinic settings and states where COVID-19 policies may have varied. Findings may not be applicable to pregnant women in their first and second trimesters, given the study population were in their third trimester of pregnancy. Social desirability bias may have influenced how participants reported their experiences, but this was reduced by anonymizing data collection. Also, this study used open-ended survey questions, which did not allow participants to be probed or to clarify their responses. Although this may have limited the depth and nuance of the qualitative data compared to those from in-depth or focus group interviews, this study was able to capture a large and diverse set of experiences during the rapidly evolving Omicron phase of the pandemic.

## 5. Conclusions

Overall, this study revealed the stressful nature of the pandemic experienced by pregnant women during the COVID-19 Omicron variant wave. Although direct and indirect pandemic factors such as pregnant women’s fears and concerns, limited social support, changes to birth plans, and unmet health-related social needs were major stressors, factors including access to social support and adaptive coping strategies acted as a stress buffer. Therefore, study findings highlight the importance of effective provider and public health communication, integrated care, and policies that will ensure the health and safety of women through pregnancy, childbirth, and the postpartum period during future public health crises.

## Figures and Tables

**Table 1:** Demographic Characteristics of Participants.

Characteristic	N	%
Race		
White	209	66.8
Black or African American	78	24.9
Multiracial	12	3.8
Asian	8	2.6
American Indian or Alaska Native	5	1.6
Native Hawaiian or Pacific Islander	1	0.3
Ethnicity		
Non-Hispanic	217	69.3
Hispanic	96	30.7
Education level		
Some High School	10	3.2
High School	113	36.1
Associate/Technical Degree	45	14.4
Bachelor’s Degree	76	24.3
Master’s Degree	45	14.4
Professional/Doctoral Degree	24	7.6
Marital status		
Single	41	13.1
Married or Committed Relationship	272	86.9
Gravida		
Primigravida	118	37.7
Multigravida	195	62.3
Pregnancy wantedness		
Not Wanted	10	3.2
Wanted Later	59	18.8
Wanted Now	244	78.0
Fully vaccinated for COVID-19		
No	146	46.6
Yes	167	53.4
Intention to be vaccinated *		
No	115	78.8
Yes	31	21.2
	Mean	SD
Age	30.4	5.5

* Total *n* = 146.

**Table 2:** Themes and Representative Participant Quotes Describing Direct COVID-19-Related Factors Affecting Maternal Stress.

Factor	Theme	Participant Quote, ID
Direct COVID-19-Related Factors Increasing Stress	Fear, Worry, and Anxiety Related to COVID-19 Infection	“I’m a nurse. The pandemic has added so much stress… [the thought of] getting COVID-19 and losing the baby has been stressful…we have had so many patients turn up with positive results and exposing us. I have a child at home.” [391]
	Fear, Worry, and Anxiety Related to Preparedness for Birth/Baby Due to Pandemic Restrictions	“…According to the new TGH policy, there will only be one support person allowed in the room. I was hoping to hire a doula for additional support to achieve an unmediated birth, but now that doesn’t seem possible and has slightly increased worry surrounding how the birth will go.” [115]
	Prevention Concerns Associated With COVID-19: Concerns Related to COVID-19 Mandates	“I feel mandates are unnecessary and create stress. I hate being forced to wear a mask when it is not actually effective. It ruins communication. I’m worried I will be forced to take an extremely uncomfortable COVID-19 test in order to not wear a mask during labor.” [393]
	Concerns Related to COVID-19 Vaccination	“Getting out of the military at the same time of my due date is stressful and being almost kicked out or reprimanded for refusing the COVID-19 vaccine while pregnant has been very stressful, as well. My workplace is a really stress induced environment.” [40]
	Lack of Social Support Due to the Pandemic	“Because of COVID-19 a lot less family and friends come around in general or even to help with my toddler when I am sick or in pain and it has been hard since many people work from home and do not like going out unless it’s necessary. This also makes it difficult to make it to certain appointments when I’m not able to drive on days I am really nauseous or in pain.” [397]
	Return to Normalcy	“Something that has increased my stress while pregnant during the COVID-19 pandemic is not knowing when everything will be back to normal and/or if this is something we will live with the rest of our lives.” [3]
Direct COVID-19-Related Factors Decreasing Stress	Preventive Measures During the COVID-19 Pandemic	“Not having to go into work/an office has been really great, especially since I was so sick the first trimester. Being able to work from home has definitely decreased stress, also in knowing I’m much less exposed [to the COVID-19 virus].” [206]
	Pandemic Coping Strategies	“Spending more time indoor with family because I get more help or spend time doing things to get distracted.” [169]

**Table 3:** Themes and Representative Participant Quotes Describing Indirect or Unrelated COVID-19 Factors Affecting Maternal Stress.

Factor	Theme	Participant Quote, ID
Indirect or Unrelated COVID-19 Factors Increasing Stress	Lack of Social Support	“I’ve had a lack of direct family support due to family being out of state, and the only family present has mental health issues that cause them to be violent. So, this has caused me at times to feel less support…” [66]
	Health-Related Social Needs	“…access to mental healthcare that is affordable has become more difficult and scarcer…Utilizing health insurance and benefits has become more difficult as the system seems to be overloaded…” [372]
	Physical Health Issues	“This is my sixth child, so my muscles are not as strong, and it hurts to walk. This is stressful when trying to work and complete tasks.” [33]
	Navigating Conflict and Grief	“Dad passed away. First grandkid and a boy which is what he always dreamed of. Bittersweet feelings. Sometimes husband doesn’t understand the time limitations and does not contribute to preparing for the journey or think things through.” [367]
Indirect or Unrelated COVID-19 Factors Decreasing Stress	Not Having to Work	“Having more time with members of my household has been helpful to decrease stress. I have taken more time being home and doing projects and home to pour into the house and the family that lives there.” [372]
	Coping Strategies	“Having a relationship with Jesus, spending time with Him in prayer, reading His Word, and knowing that others are praying for me.” [392]
	Social Support	“Knowing that my mom is doing everything she can to keep me happy, and my siblings and boyfriend make sure I have everything I want and need even when I say I don’t need it or want it.” [272]

## Data Availability

The data presented in this study are available from the corresponding author upon reasonable request. Due to ethical and privacy restrictions, the data cannot be made publicly accessible.

## References

[1] World Health Organization (2020). WHO Director-General’s Opening Remarks at the Media Briefing on COVID-19. https://www.who.int/news-room/speeches/item/who-director-general-s-opening-remarks-at-the-media-briefing-on-covid-19---11-march-2020.

[2] Nicola M., Alsafi Z., Sohrabi C., Kerwan A., Al-Jabir A., Iosifidis C., Agha M., Agha R. (2020). The socio-economic implications of the coronavirus pandemic (COVID-19): A review. Int. J. Surg..

[3] Ferwana I., Varshney L.R. (2024). The impact of COVID-19 lockdowns on mental health patient populations in the United States. Sci. Rep..

[4] Javaid S., Barringer S., Compton S.D., Kaselitz E., Muzik M., Moyer C.A. (2021). The impact of COVID-19 on prenatal care in the United States: Qualitative analysis from a survey of 2519 pregnant women. Midwifery.

[5] Meaney S., Leitao S., Olander E.K., Pope J., Matvienko-Sikar K. (2022). The impact of COVID-19 on pregnant womens’ experiences and perceptions of antenatal maternity care, social support, and stress-reduction strategies. Women Birth.

[6] Moyer C.A., Compton S.D., Kaselitz E., Muzik M. (2020). Pregnancy-related anxiety during COVID-19: A nationwide survey of 2740 pregnant women. Arch. Womens Ment. Health.

[7] Arzamani N., Soraya S., Hadi F., Nooraeen S., Saeidi M. (2022). The COVID-19 pandemic and mental health in pregnant women: A review article. Front. Psychiatry.

[8] Puertas-Gonzalez J.A., Mariño-Narvaez C., Romero-Gonzalez B., Vilar-López R., Peralta-Ramirez M.I. (2023). Resilience, stress and anxiety in pregnancy before and throughout the pandemic: A structural equation modelling approach. Curr. Psychol..

[9] Zilver S.J.M., Broekman B.F.P., Hendrix Y.M.G.A., De Leeuw R.A., Mentzel S.V., Van Pampus M.G., De Groot C.J.M. (2021). Stress, anxiety and depression in 1466 pregnant women during and before the COVID-19 pandemic: A Dutch cohort study. J. Psychosom. Obstet. Gynecol..

[10] Preis H., Mahaffey B., Heiselman C., Lobel M. (2020). Vulnerability and resilience to pandemic-related stress among U.S. women pregnant at the start of the COVID-19 pandemic. Soc. Sci. Med..

[11] King L.S., Feddoes D.E., Kirshenbaum J.S., Humphreys K.L., Gotlib I.H. (2023). Pregnancy during the pandemic: The impact of COVID-19-related stress on risk for prenatal depression. Psychol. Med..

[12] Thayer Z.M., Gildner T.E. (2020). COVID-19-related financial stress associated with higher likelihood of depression among pregnant women living in the United States. Am. J. Hum. Biol..

[13] Sun S., Luo C., Zeng X., Wu Q. (2024). The relationship between pregnancy stress and mental health of the pregnant women: The bidirectional chain mediation roles of mindfulness and peace of mind. Front Psychol..

[14] Biaggi A., Conroy S., Pawlby S., Pariante C.M. (2016). Identifying the women at risk of antenatal anxiety and depression: A systematic review. J. Affect. Disord..

[15] Dennis C.L., Falah-Hassani K., Shiri R. (2017). Prevalence of antenatal and postnatal anxiety: Systematic review and meta-analysis. Br. J. Psychiatry.

[16] Yin X., Sun N., Jiang N., Xu X., Gan Y., Zhang J., Qiu L., Yang C., Shi X., Chang J. (2021). Prevalence and associated factors of antenatal depression: Systematic reviews and meta-analyses. Clin. Psychol. Rev..

[17] Tomfohr-Madsen L.M., Racine N., Giesbrecht G.F., Lebel C., Madigan S. (2021). Depression and anxiety in pregnancy during COVID-19: A rapid review and meta-analysis. Psychiatry Res..

[18] Fan S., Guan J., Cao L., Wang M., Zhao H., Chen L., Yan L. (2021). Psychological effects caused by COVID-19 pandemic on pregnant women: A systematic review with meta-analysis. Asian J. Psychiatr..

[19] Sun F., Zhu J., Tao H., Ma Y., Jin W. (2021). A systematic review involving 11,187 participants evaluating the impact of COVID-19 on anxiety and depression in pregnant women. J. Psychosom. Obstet. Gynecol..

[20] Chin K., Wendt A., Bennett I.M., Bhat A. (2022). Suicide and Maternal Mortality. Curr. Psychiatry Rep..

[21] Araji S., Griffin A., Dixon L., Spencer S.K., Peavie C., Wallace K. (2020). An Overview of Maternal Anxiety During Pregnancy and the Post-Partum Period. J. Ment. Health Clin. Psychol..

[22] van Dijk M.T., Talati A., Barrios P.G., Crandall A.J., Lugo-Candelas C. (2024). Prenatal depression outcomes in the next generation: A critical review of recent DOHaD studies and recommendations for future research. Semin. Perinatol..

[23] Jin Y., Murray L. (2023). Perinatal mental health and women’s lived experience of the COVID-19 pandemic: A scoping review of the qualitative literature 2020–2021. Midwifery.

[24] Aydin R., Aktaş S. (2021). An investigation of women’s pregnancy experiences during the COVID-19 pandemic: A qualitative study. Int. J. Clin. Pract..

[25] Jones K., Harrison V., Moulds M.L., Lazard L. (2022). A qualitative analysis of feelings and experiences associated with perinatal distress during the COVID-19 pandemic. BMC Pregnancy Childbirth.

[26] Keating N.E., Dempsey B., Corcoran S., McAuliffe F.M., Lalor J., Higgins M.F. (2021). Women’s experience of pregnancy and birth during the COVID-19 pandemic: A qualitative study. Irish J. Med. Sci..

[27] Kolker S., Biringer A., Bytautas J., Blumenfeld H., Kukan S., Carroll J.C. (2021). Pregnant during the COVID-19 pandemic: An exploration of patients’ lived experiences. BMC Pregnancy Childbirth.

[28] Mirzakhani K., Shoorab N.J., Akbari A., Khadivzadeh T. (2022). High-risk pregnant women’s experiences of the receiving prenatal care in COVID-19 pandemic: A qualitative study. BMC Pregnancy Childbirth.

[29] Williams C.E., Berkowitz D., Rackin H.M. (2023). Exploring the experiences of pregnant women in the U.S. during the first year of the COVID-19 pandemic. J. Soc. Issues.

[30] Kinser P., Jallo N., Moyer S., Weinstock M., Barrett D., Mughal N., Stevens L., Rider A. (2022). “It’s always hard being a mom, but the pandemic has made everything harder”: A qualitative exploration of the experiences of perinatal women during the COVID-19 pandemic. Midwifery.

[31] Ashby G.B., Riggan K.A., Huang L., Torbenson V.E., Long M.E., Wick M.J., Allyse M.A., Rivera-Chiauzzi E.Y. (2022). “I had so many life-changing decisions I had to make without support”: A qualitative analysis of women’s pregnant and postpartum experiences during the COVID-19 pandemic. BMC Pregnancy Childbirth.

[32] Barbosa-Leiker C., Smith C.L., Crespi E.J., Brooks O., Burduli E., Ranjo S., Carty C.L., Hebert L.E., Waters S.F., Gartstein M.A. (2021). Stressors, coping, and resources needed during the COVID-19 pandemic in a sample of perinatal women. BMC Pregnancy Childbirth.

[33] Ali S., Giovanetti M., Johnston C., Urdaneta-Páez V., Azarian T., Cella E. (2024). From Emergence to Evolution: Dynamics of the SARS-CoV-2 Omicron Variant in Florida. Pathogens.

[34] Lazarus R., Folkman S. (1984). Stress, Appraisal, and Coping.

[35] Cohen S., Wills T.A. (1985). Stress, Social Support, and the Buffering Hypothesis. Psychol. Bull..

[36] Reid C.N. Psychosocial Factors Influencing the Mental Health and Maternal-Fetal Attachment in Pregnant Women During the COVID-19 Pandemic. PHD Dissertation, University of South Florida, Tampa, FL, USA, 2023. https://www.proquest.com/dissertations-theses/psychosocial-factors-influencing-mental-health/docview/2901427017/se-2?accountid=14745.

[37] Braun V., Clarke V. (2006). Using thematic analysis in psychology. Qual. Res. Psychol..

[38] O’Connor C., Joffe H. (2020). Intercoder Reliability in Qualitative Research: Debates and Practical Guidelines. Int. J. Qual. Methods.

[39] Bernard H.R., Ryan G.W. (2010). Analyzing Qualitative Data Systematic Approaches.

[40] Saldana J. (2015). The Coding Manual for Qualitative Researchers.

[41] Hennink M., Kaiser B.N. (2022). Sample sizes for saturation in qualitative research: A systematic review of empirical tests. Soc. Sci. Med..

[42] SAS Institute Inc. (2016). SAS Software.

[43] Lebel C., MacKinnon A., Bagshawe M., Tomfohr-Madsen L., Giesbrecht G. (2020). Elevated depression and anxiety symptoms among pregnant individuals during the COVID-19 pandemic. J. Affect. Disord..

[44] Liu M., Li N., Cai X., Feng X., Wang R., Xiong P. (2021). The Prevalence of Psychological Symptoms in Pregnant Healthcare Workers (HCWs) and Pregnant Non-HCWs During the Early Stage of COVID-19 Pandemic in Chongqing, China. Front. Psychiatry.

[45] Ataman H., Tuncer M. (2023). The effect of COVID-19 fear on prenatal distress and childbirth preference in primipara. Rev. Assoc. Med. Bras..

[46] Giesbrecht G.F., Rojas L., Patel S., Kuret V., MacKinnon A.L., Tomfohr-Madsen L., Lebel C. (2021). Fear of COVID-19, mental health, and pregnancy outcomes in the pregnancy during the COVID-19 pandemic study: Fear of COVID-19 and pregnancy outcomes. J. Affect. Disord..

[47] Ayers S., Meades R., Sinesi A., Cheyne H., Maxwell M., Best C., McNicol S., Alderdice F., Jomeen J., Shakespeare J. (2025). COVID-19 and anxiety in pregnancy and postpartum: A longitudinal survey. BMC Public Health.

[48] Preis H., Mahaffey B., Heiselman C., Lobel M. (2021). The impacts of the COVID-19 pandemic on birth satisfaction in a prospective cohort of 2,341 U.S. women. Women Birth.

[49] Breman R.B., Neerland C., Bradley D., Burgess A., Barr E., Burcher P. (2021). Giving birth during the COVID-19 pandemic, perspectives from a sample of the United States birthing persons during the first wave: March-June 2020. Birth.

[50] Tan A., Blair A., Homer C.S.E., Digby R., Vogel J.P., Bucknall T. (2024). Pregnant and postpartum women’s experiences of the indirect impacts of the COVID-19 pandemic in high-income countries: A qualitative evidence synthesis. BMC Pregnancy Childbirth.

[51] Matvienko-Sikar K., Pope J., Cremin A., Carr H., Leitao S., Olander E.K., Meaney S. (2021). Differences in levels of stress, social support, health behaviours, and stress-reduction strategies for women pregnant before and during the COVID-19 pandemic, and based on phases of pandemic restrictions, in Ireland. Women Birth.

[52] Dewi A., Safaria T., Supriyatiningsih S., Dewi D.T.K. (2023). Efforts and expectations of pregnant women against the impact of the COVID-19 pandemic: A phenomenological study. BMC Pregnancy Childbirth.

[53] Comparcini D., Tomietto M., Pastore F., Nichol B., Miniscalco D., Flacco M.E., Stefanizzi P., Tafuri S., Cicolini G., Simonetti V. (2024). Factors Influencing COVID-19 Vaccine Hesitancy in Pregnant and Breastfeeding/Puerperium Women: A Cross-Sectional Study. Vaccines.

[54] Skirrow H., Barnett S., Bell S., Riaposova L., Mounier-Jack S., Kampmann B., Holder B. (2022). Women’s views on accepting COVID-19 vaccination during and after pregnancy, and for their babies: A multi-methods study in the UK. BMC Pregnancy Childbirth.

[55] Casubhoy I., Kretz A., Tan H.L., St Clair L.A., Parish M., Golding H., Bersoff-Matcha S.J., Pilgrim-Grayson C., Berhane L., Pekosz A. (2024). A scoping review of global COVID-19 vaccine hesitancy among pregnant persons. Npj Vaccines.

[56] Peters M.D.J. (2022). Addressing vaccine hesitancy and resistance for COVID-19 vaccines. Int. J. Nurs. Stud..

[57] Kokkinaki T., Hatzidaki E. (2022). COVID-19 Pandemic-Related Restrictions: Factors That May Affect Perinatal Maternal Mental Health and Implications for Infant Development. Front. Pediatr..

[58] Al-Mutawtah M., Campbell E., Kubis H.P., Erjavec M. (2023). Women’s experiences of social support during pregnancy: A qualitative systematic review. BMC Pregnancy Childbirth.

[59] Lalor J.G., Sheaf G., Mulligan A., Ohaja M., Clive A., Murphy-Tighe S., Ng E.D., Shorey S. (2022). Parental experiences with changes in maternity care during the COVID-19 pandemic: A mixed-studies systematic review. Women Birth.

[60] Asselmann E., Kunas S.L., Wittchen H.U., Martini J. (2020). Maternal personality, social support, and changes in depressive, anxiety, and stress symptoms during pregnancy and after delivery: A prospective-longitudinal study. PLoS ONE.

[61] Wheeler J.M., Misra D.P., Giurgescu C. (2021). Stress and coping among pregnant black women during the COVID-19 pandemic. Public Health Nurs..

[62] Lafarga Previdi I., Hernández Otero N., Guzzi Vasques A., Ayala I., Alvelo Colón G., Guilloty N., Medina J., Cancel-Garcia M., Cordero J., Alshawabkeh A.N. (2025). The Impact of COVID-19 on Maternal Health: Quantitative Data Related to Risk and Protective Factors Among Pregnant and Postpartum Women in Puerto Rico. Int. J. Environ. Res. Public Health.

[63] Cigaran R.G., Peltecu G., Mustata L.M., Botezatu R. (2024). Stress Coping Strategies of Pregnant Women during COVID-19 Pandemic: A Literature Review. Maedica A J. Clin. Med..

[64] Khoury J.E., Atkinson L., Bennett T., Jack S.M., Gonzalez A. (2021). Coping strategies mediate the associations between COVID-19 experiences and mental health outcomes in pregnancy. Arch. Womens Ment. Health.

[65] Burgess A., Breman R.B., Roane L.A., Dada S., Bradley D., Burcher P. (2022). Impact of COVID-19 on pregnancy worry in the United States. Birth.

[66] Goodsmith N., Ijadi-Maghsoodi R., Melendez R.M., Dossett E.C. (2020). Addressing the Urgent Housing Needs of Vulnerable Women in the Era of COVID-19: The Los Angeles County Experience. Psychiatr. Serv..

[67] Versey H.S., Russell C.N. (2023). The impact of COVID-19 and housing insecurity on lower-income Black women. J. Soc. Issues.

[68] Masters G.A., Asipenko E., Bergman A.L., Person S.D., Brenckle L., Simas T.A.M., Ko J.Y., Robbins C.L., Byatt N. (2021). Impact of the COVID-19 pandemic on mental health, access to care, and health disparities in the perinatal period. J. Psychiatr. Res..

[69] Pope J., Olander E.K., Leitao S., Meaney S., Matvienko-Sikar K. (2022). Prenatal stress, health, and health behaviours during the COVID-19 pandemic: An international survey. Women Birth.

[70] Eleftheriades M., Vousoura E., Eleftheriades A., Pervanidou P., Zervas I.M., Chrousos G., Vlahos N.F., Sotiriadis A. (2022). Physical Health, Media Use, Stress, and Mental Health in Pregnant Women during the COVID-19 Pandemic. Diagnostics.

[71] McGowan L., Astuti A., Hafidz F., Pratiwi C., Yulian V., Hughes E., Pratiwi A., Indomo E.N., Fu Y. (2023). The effect of COVID-19 on women’s experiences of pregnancy, birth and postpartum in Indonesia: A rapid online survey. BMC Pregnancy Childbirth.

[72] Liu C.H., Erdei C., Mittal L. (2021). Risk factors for depression, anxiety, and PTSD symptoms in perinatal women during the COVID-19 Pandemic. Psychiatry Res..

[73] Liu J., Hung P., Alberg A.J., Hair N.L., Whitaker K.M., Simon J., Taylor S.K. (2021). Mental health among pregnant women with COVID-19–related stressors and worries in the United States. Birth.

[74] Savvoudi D.M., Orovou E., Dagla M., Kirkou G., Iatrakis G., Antoniou E. (2024). Domestic Violence in Pregnancy during the Pandemic Era: A Systematic Review. Mædica.

[75] Ruyak S.L., Kivlighan K.T. (2021). Perinatal Behavioral Health, the COVID-19 Pandemic, and a Social Determinants of Health Framework. J. Obstet. Gynecol. Neonatal Nurs..

[76] Berthelot N., Garon-Bissonnette J., Drouin-Maziade C., Bergeron V., Sériès T. (2023). STEP-COVID: A pilot study of a prenatal intervention for pregnant women during the COVID-19 pandemic. Sci. Rep..

[77] Naja S., Elyamani R., Chehab M., Ali Siddig Ahmed M., Babeker G., Lawand G., Singh R., Adli N., Mohamad T., Bougmiza I. (2023). The impact of telemental health interventions on maternal mental health outcomes: A pilot randomized controlled trial during the COVID-19 pandemic. Health Psychol. Behav. Med..

[78] Ahmed S.K. (2024). The pillars of trustworthiness in qualitative research. J. Med. Surgery Public. Health.

